# Self-Harm, Suicidal Ideation and Attempts among School-Attending Adolescents in Bamako, Mali

**DOI:** 10.3390/children9040542

**Published:** 2022-04-11

**Authors:** Wu Yedong, Souleymane Papa Coulibaly, Aissata Mahamadou Sidibe, Thérèse Hesketh

**Affiliations:** 1 Center for Global Health, Zhejiang University, Hangzhou 310058, China; wuyedong@zju.edu.cn (W.Y.); a.sidibe@zju.edu.cn (A.M.S.); 2 Faculty of Medicine and Odontostomatogy, University of Technical Sciences and Technologies, Bamako 00223, Mali; sp.coulibaly@fmos.usttb.edu.ml; 3 The Institute for Global Health, University College London, London WC1N1EH, UK

**Keywords:** adolescence, self-harm, suicide, suicide ideation, suicide attempts, depression, anxiety, adolescents, Mali

## Abstract

Suicide and self-harm are major public health concerns for adolescents globally, but there is a dearth of related research in West Africa. This study aims to examine the prevalence and associated factors for self-harm, suicidal ideation and suicide attempts among adolescents in the West African country of Mali. A questionnaire survey was conducted among adolescents attending school or university in August 2019 in Bamako, the capital of Mali. Logistical constraints necessitated convenience sampling. Outcome measures were self-harm and suicide ideation and attempts. Predictor variables included sociodemographic characteristics, bullying and mental health problems. There were 606 respondents who completed questionnaires; their mean age was 16.1 (SD = 2.4); 318 (52.5%) were identified as male; and 44.4% reported self-harm at some point in their life, with 21% reporting suicide ideation and 9.7% actual suicide attempts. For all three outcomes, older age, knowing somebody personally who had experienced self-harm or taken their own life, moderate to severe depression or anxiety, and being a victim of bullying were highly significant risk factors for self-harm, suicidal ideation and suicide attempts in these adolescents, while high self-esteem decreased the risk. The study suggests that self-harm and suicidal behaviour are relatively common in Malian adolescents who are still in education. However, much more research is needed to better understand this phenomenon.

## 1. Introduction

Suicide was the fourth leading cause of death among 15–19-year-olds globally [1] and more than 90% of adolescent suicides take place in low- and middle-income countries (LMICs) [2]. In many settings, suicide is highly stigmatized and thus under-reported, so routine data almost certainly underestimate the magnitude of the problem. To address the problem requires an understanding of the prevalence and associated risk factors for suicide. Two key measures are suicide attempts and suicide ideation, both of which require detailed survey data to estimate, since they are not reported in routine data. No standard definitions of suicide-related terms have been agreed. In this study, suicide attempt (SA) refers to self-directed, non-fatal, potentially injurious behavior with an intent to die. Suicidal ideation (SI) refers to thoughts of engaging in suicide-related behavior [3,4]. There is evidence of a relationship between suicide ideation and attempts; the results of a study among US adolescents showed that one-third (33.9%) of suicidal ideators went on to make an attempt [5].

Self-harm is often considered as part of this continuum but this phenomenon is generally regarded as unrelated to suicide, since there is no intent to die. It generally refers to intentional self-injury, such as self-laceration, self-battering, taking overdoses or exhibiting deliberate recklessness [3]. However, self-harm may lead to death, often accidentally, with 67,000 adolescent deaths estimated globally in 2015 [6].

Understanding and identifying the relevant risk factors is a priority in developing effective prevention and treatment programmes. Several factors, such as sociodemographic and educational factors, individual negative life events and family adversity, and psychiatric and psychological factors, have been found to be associated with self-harm and suicide in adolescents across a range of settings [7]. However, the overwhelming majority of the large literature about self-harm and suicide behaviors among adolescent populations comes from high income countries and much less is known about the situation in sub-Saharan Africa (SSA). Suicide attempts are reported in routine data in some countries (though the validity and reliability of the data are questionable), but they are not reported specifically in West Africa [8,9]. According to a recent meta-analysis [10], the global lifetime prevalence of suicidal ideation, suicide attempts and self-harm were 18%, 6% and 14%, respectively, in children and adolescents between 1989 and 2018. It showed that suicidal and self-harm behavior was higher in children and adolescents who were full-time school attendees and in those living in developing countries. Analysis by continent showed that the lifetime prevalence of SI and the 12-month prevalence of SA and SI were highest in Africa. A review of suicidal behaviours among school-going adolescents from studies in 32 low- and middle-income countries showed that the highest prevalence was in sub-Saharan Africa (SSA) [11]. However, the majority of the studies included in such reviews have been conducted in South Africa; there are very few from West Africa [12]. Moreover, there is no other research into self-harm and suicidal phenomena in adolescents in Mali.

Mali is a landlocked low-income Muslim country in West Africa with a population of around 20 million, of which 24% are adolescents aged 10–19 [13]. It is one of the poorest countries in the world, ranking 184 on the Human Development Index [14]. The vast majority of Malians live in the southern region, including the capital, Bamako, which has 2.7 million inhabitants. Mali has been experiencing insecurity and instability since the military coup of 2012 with the northern and central regions occupied by armed groups [15]. School enrollment in secondary education in Mali is 41.0% overall (37.0% of females and 45.0% of males) and in tertiary education 5.5% (3.2% of females and 7.7% of males) [16]. In addition, recent government coups have challenged an already failing political system. Prospects for youth are bleak given a deteriorating educational system and very high youth unemployment.

There are increasing concerns about mental health problems in Mali. However, little is known about the prevalence of mental health disorders in the general Malian population and especially among adolescents [17].

This aims of this study, therefore, are: (1) to explore the prevalence and risk factors for self-harm and suicide ideation and attempts in school-attending Malian adolescents; and (2) to explore the relationship between self-harm, SI and SA, and measures of depression and anxiety.

## 2. Materials and Methods

### 2.1. Sample

This cross-sectional study was carried out in a population of 729 students aged 10–20 years old in August, 2019 in Bamako, the capital of Mali. In Mali, the standard entry age of middle school/high school/tertiary education is 13/16/19 years old. The upper age varies considerably due to late entry into school, repetition of years because of failure and temporary dropout [18]. Self-reported questionnaires were completed randomly at 3 middle schools, 5 high schools and among freshmen at 2 universities in 5 neighborhoods representing the socio-economic differences across the city. The permission of all the principals was obtained. In the middle and high schools, every student present on the day was invited to participate and classes were the sampling units, though class size varied considerably. In the two universities, we used convenience sampling of first year students, who were approached directly. We aimed to include equal numbers of males and females. Members of the research team described the aim of the study and explained how to complete the questionnaire.

### 2.2. Measures

The questionnaire consisted of questions about socio-demographic characteristics, self-evaluation of health and academic performance, bullying in school, key measures of mental health (depression, anxiety and self-esteem) and three crucial dependent variables, experience of self-harm, suicide ideation and suicide attempts, as described in the Introduction. The questionnaire was translated from English into French, the official language in Mali, and was revised by a team of three adolescents, two local psychologists and a professional translator.

Bullying in school was measured by asking “Have you ever been bullied at school?” and “Have you ever bullied others at school?”. The response options were “Never”, “Occasionally”, “Sometimes”, “Frequently” and “Don’t know”.

Depression was assessed using the 10-item Center for Epidemiologic Studies Depression Scale (CESD-10), which has been widely used in many settings [19,20,21]. Questions refer to symptoms in the past 7 days and are answered on a 4-point Likert scale, ranging from “On less than 1 day” (score of 0) to “On 5–7 days” (score of 3), with a total score range of 0 to 30. A cut-off score of 10 or more was used to indicate moderate and severe depressive symptoms, as in previous studies in SSA settings [22,23,24,25,26].

Anxiety was assessed with the widely used 7-item Generalized Anxiety Disorder Scale (GAD-7) [27]. The items were rated on a 4-point Likert scale, ranging from 0 (not at all) to 3 (nearly always), with total scores ranging from 0 to 21. A cut-off score of 10 and above indicated moderate and severe anxiety [27,28].

Self-esteem was measured using the 10-item Rosenberg Self-Esteem Scale (RSES) [29]. It uses a 4-point Likert scale ranging from 1 (strongly agree) to 4 (strongly disagree), with a score 25 or higher indicating high self-esteem and a score lower than 25 indicating low self-esteem [30].

Self-harm was assessed by asking “Have you ever intentionally injured yourself?” and “Have you ever experienced self-harm?”. Suicidal ideation (SI) was assessed by asking “Have you ever thought about taking your own life?”. Suicide attempt (SA) was measured by asking “Have you ever attempted to take your own life?”. Response options for all questions were “Never”, “Occasionally”, “Sometimes”, “Frequently” and “Don’t know”.

### 2.3. Ethics

Ethical approvals for the study were granted by the Ethics Committee at Zhejiang University in China (Approval Code: ZGL201906-3, Approval Date: 18 June 2019) as well as the ethics board of the University of Sciences, Techniques and Technologies of Bamako (USTTB) in Mali (Approval Code: No2019/96/CE/FMPOS, Approval Date: 21 August 2019). The participants were informed that participation was voluntary, that they did not need to answer any questions they felt uncomfortable with and that they could withdraw at any time. All the participants provided verbal informed consent and were assured that all data would be anonymous and confidential.

### 2.4. Statistical Analysis

Descriptive statistics were used for sociodemographic variables (age, school level, religion, education level of parents, polygamous father, number of father’s children, currently living with parents and family economic status). To examine the independent impact of all the predictors, especially the variables (religion, education level of mother/father, polygamous father, number of father’s children) within the Malian context, regression models were calculated for each independent variable, respectively, with all the outcome variables (self-harm, SI and SA) adjusted for sex, age and family economic status. Analyses were conducted using SPSS version 26.0 for Windows.

## 3. Results

Questionnaires with missing responses to key variables were excluded from analyses. Thus, a total of 606 completed questionnaires were eligible for analysis—a response rate of 83.1%.

### 3.1. Sociodemographic Characteristics 

These are shown in Table 1. The age range of respondents was 10 to 20, with a mean age of 16.1 (SD = 2.4); 318 (52.5%) identified as male. Among the respondents, 173 (28.5%) were middle school students, 300 (49.5%) were high school students and 133 (21.9%) were in their first year at university; the overwhelming majority (537, 88.6%) self-identified as Muslim, 60 (9.9%) as Christian; and 451 (74.4%) were living with both parents. The main reason for not living with parents was to be close to the school or university they attended (32%). The average number of children per family was 5.6, and 22% of fathers were in polygamous relationships).

### 3.2. Health and Mental Health

These are shown in Table 2. Most participants reported that they were in good health (60.2%) and that they achieved good academic performance (61.6%). Out of all the respondents, 295 (48.7%) students had scores indicating moderate to severe depression, 123 (20.3%) students showed moderate to severe anxiety and 101 (16.7%) reported scores indicating low self-esteem. One-third of participants had been bullied (36.6%) and a quarter admitted to bullying others (26.7%). Males (101, 31.7%) were more likely to be bullies than females (61, 21.2%).

### 3.3. Self-Harm, Suicidal Ideation and Suicide Attempts

These are shown in Table 3. Nearly half of the students (269 (44.4%)) stated that they had experienced self-harm, 127 (21.0%) had thought about taking their own life, while 59 (9.7%) had attempted to take their own life. There were no significant sex differences for all three behaviours. Among the respondents, 233 (38.5%) personally knew somebody who had experienced self-harm.

### 3.4. Predictors for Self-Harm

These are shown in Table 4. Before adjustment, older age (*p* < 0.001), poorer health (*p* < 0.001), poorer academic performance (*p* < 0.001), moderate to severe depression (*p* < 0.001), moderate to severe anxiety (*p* < 0.001), knowing somebody personally who had experienced self-harm (*p* < 0.001) and having been bullied at school (*p* < 0.001) significantly increased the risk of self-harm. After adjusting for the confounders (age, sex and family economic status), older students were almost four times as likely to experience self-harm as those aged 10–14 years. Knowing somebody personally who had self-harmed increased the likelihood of self-harm six-fold. Moderate to severe anxiety increased the risk of self-harm three-fold and moderate to severe depression doubled the risk. Poorer health and being a victim of bullying also doubled the risk of self-harm.

### 3.5. Predictors for Suicidal Ideation 

These are shown in Table 4. Before adjustment, poorer health (*p* < 0.001), poorer academic performance (*p* < 0.01), moderate to severe depression (*p* < 0.001), moderate to severe anxiety (*p* < 0.001), knowing somebody personally who had self-harmed (*p* < 0.001), knowing somebody personally who had taken their own life (*p* < 0.01) and having been bullied at school (*p* < 0.01) all significantly increased the risk of SI. After adjustment, moderate to severe anxiety and knowing somebody personally who had experienced self-harm and suicide both increased the likelihood of SI by almost three-fold. Poorer health and academic performance, moderate to severe depression and having been bullied at school doubled the risk of SI. In addition, high self-esteem significantly decreased the risk of SI after adjustment (*p* < 0.01).

### 3.6. Predictors for Suicide Attempts

These are shown in Table 4. Before adjustment, poorer health (*p* = 0.010), knowing somebody personally who had taken their own life (*p* < 0.001) and having been bullied at school (*p* < 0.01) significantly increased the risk of SA. After adjusting for the confounding variables, participants who knew somebody personally who had taken their own life were six times more likely to have attempted suicide. Having been bullied at school doubled the likelihood of SA. High self-esteem decreased the likelihood of SA after adjustment (*p* < 0.05).

## 4. Discussion

To our knowledge, this is the first study to explore self-harm and suicide behaviours among school-attending adolescents in Mali. Our estimates show that self-harm, SI and SA are common among adolescents in Bamako. Nearly half of the participants (44.4%) reported self-harm at some point in their life, with 21% reporting suicide ideation and 9.7% actual suicide attempts. How these figures relate to completed suicides in this population is unclear, not least because there are no accurate records or even estimates. One study showed that, of 547 suicidal poisoning cases recorded in hospitals in Southern Mali between 2000 and 2010, 62% were 15–24-year-olds [31], suggesting that adolescents are an especially vulnerable age group.

In terms of self-harm, our findings are much higher than both the global estimate for full-time school-attending adolescents, which is reported at 15.3% [10], and the median lifetime prevalence of 10.3% in 18 sub-Saharan African countries reported in a recent review [12]. The authors of the latter concluded that self-harm is emerging as a public health challenge among young people in sub-Saharan Africa [12]. Another study reported a rate of 47% for 17–21-year-olds attending university in South Africa [32]. There are few other studies from West Africa, but one from Ghana showed a prevalence of 22.0% among school adolescents [33]. There are a number of explanations for these disparities, including underlying socio-cultural differences and a lack of consistency in defining and assessing self-harming behaviour. This of course makes comparisons across countries very difficult. Further research is needed to determine precisely what is understood by self-harm in different cultures. For example, a qualitative study in Ghana found that some adolescents used self-harm as a protest against powerlessness, harsh punishment and abuse by their family [34]. This was also informally reported by several Malian students when interviewed informally after this survey.

In terms of SI and SA, one fifth of these adolescents had thought about taking their own life and almost one tenth had attempted suicide. This is close to the global lifetime prevalence of SI (19.5%) but higher than the global lifetime prevalence of SA (6.7%) among full-time school-attending adolescents [10]. These figures are higher than the lifetime prevalence rates of suicidal ideation and suicide attempts, which were found to be 9% and 3% among 13–19-year-old students [35].

Our findings highlight a number of points.

Firstly, no significant sex differences were found for the rates of self-harm, SI or SA, as other studies have shown [35,36,37]. This may seem counterintuitive given that Mali is a largely patriarchal society, where women have traditionally had fewer rights and where professional and personal expectations for young girls are often limited to marrying and producing children. Previous research in Mali showed a higher rate of suicidal behaviours in women, resulting from socially and religiously sanctioned oppression, gender role discrimination or norms such as early and forced marriage and exclusion from school [31,38]. However, all the females in our sample were living in the capital, attending school and university, and thus represented an elite of Malian females who have continued their education into late adolescence and are therefore not representative of all Malian girls [39].

Secondly, the consistent strong risk factors for self-harm, SI and SA were age, personally knowing people who had experienced self-harm or who had taken their own life, and being a victim of bullying. We found that the prevalence of self-harm, SI and SA increased with age across our sample. Possible explanations are the stressful college entrance examination, the sometimes difficult transition to university life, which has been observed as an important vulnerability factor in other populations [40], and the anxiety related to high youth unemployment which currently stands at (40%) in Mali [14], with a higher rate among university graduates. This is referred to locally as thé chômeur (which translates as the unemployed tea drinker) [41,42]. Knowing somebody personally who had experienced self-harm or suicide is a consistent risk factor for self-harm, SI and SA. Such imitative behaviour has been described as a form of social contagion. There are concerns in many countries about increases in such imitation, often driven by social media [43,44]. While it is unclear precisely how many adolescents in Bamako have a smartphone, it is reported that 38% of Bamako residents across all age groups use smartphones, with the young and well-educated using them the most [45]. Exposure to negative messaging and images encouraging imitative behaviour may have greater effects on the more vulnerable. Being a victim of bullying is the third consistent risk factor. This aligns with other studies. Having been bullied at school was positively associated with self-harm, SI and SA among school-ongoing students in other African countries, including Ghana, Benin, Uganda and Malawi [33,46,47,48,49,50]. This finding is also consistent with a global study that bullying victimization is associated with suicide attempts among school children [51].

Thirdly, as observed elsewhere, depression and anxiety play an important role in the prediction of self-harm, SI and SA [7,35,52]. Clearly, the predictors mentioned above, such as unemployment and exam stress, may lead to depression and anxiety which then results in self-harm and suicidal behaviour. High self-esteem decreased the risk of suicidal thoughts and suicide attempts, as observed elsewhere [35,53].

### Limitations

Our findings should be interpreted in light of several limitations. First, this study was only conducted in the capital city using convenience sampling because of logistical constraints. Only school-attending adolescents were included, so the sample is only broadly representative of Bamako adolescents in education. In addition, the sample size is small, limiting the validity of comparisons across sub-groups, especially in relation to schools, between which there were large differences in sample size. Secondly, only one question was used for self-harm, SI and SA, without any definitions provided, as has been the practice in similar studies. Self-harm, in particular, may have been interpreted in different ways by the respondents, thus affecting overall estimates. In addition, the structured questionnaire did not allow for exploration of the reasons for self-harm, SI and SA. Thirdly, given the lack of existing studies in Mali, cut-off points for anxiety, depression and self-esteem drew on international studies which may not be appropriate for Mali, given its unique social, cultural, educational and economic factors. This may also challenge the validity of comparisons with other countries. Fourthly, self-reporting is likely to lead to social desirability bias, especially given the sensitivity of the topics and the taboo against self-harm and suicide behaviors which are common in some Malian ethnic groups [54,55]. Finally, the cross-sectional methodology does not permit causal interpretation of the findings [56].

## 5. Conclusions

This exploratory study found a high prevalence of self-harm and suicidal behaviours among adolescents in Bamako, Mali. The evidence of the current study highlights the need for further research with larger samples and across a range of different settings to better understand these phenomena among Malian adolescents.

## Figures and Tables

**Table 1:** Sociodemographic characteristics of participants by sex.

Variables	Male (*n* = 318, 52.5 %)	Female (*n* = 288, 47.5%)	Total (*n* = 606)
Age (mean, s.d.) ^a^	16.1 (2.5)	16.0 (2.3)	16.1 (2.4)
Age			
10–12	30 (9.4)	22 (7.6)	52 (8.6)
13–15	98 (30.8)	90 (31.3)	188 (31.0)
16–18	109 (34.3)	128 (44.4)	237 (39.1)
19–20	81 (25.5)	48 (16.7)	129 (21.3)
School level (age)			
Middle school students (10–19)	98 (30.8)	75 (26.0)	173 (28.5)
High school students (13–20)	142 (44.7)	158 (54.9)	300 (49.5)
Freshmen (17–20)	78 (24.5)	55 (19.1)	133 (21.9)
Religion			
No religion	1 (0.3)	0 (0.0)	1 (0.2)
Islam	282 (88.7)	255 (88.5)	537 (88.6)
Christian	31 (9.7)	29 (10.1)	60 (9.9)
Education level of father			
No education	19 (6.0)	9 (3.1)	28 (4.6)
Madrassa/Madrasah	34 (10.7)	29 (10.1)	63 (10.4)
Primary/middle school	58 (18.2)	58 (20.1)	116 (19.1)
Higher education	134 (42.1)	123 (42.7)	257 (42.4)
Education level of mother			
No education	36 (11.3)	30 (10.4)	66 (10.9)
Madrassa/Madrasah	39 (12.3)	32 (11.1)	71 (11.7)
Primary/middle school	78 (24.5)	76 (26.4)	154 (25.4)
Higher education	83 (26.1)	97 (33.7)	180 (29.7)
Polygamous father	80 (25.2)	54 (18.8)	134 (22.1)
Number of father’s children (mean, s.d.) ^a^	5.8 (2.9)	5.4 (2.4)	5.6 (2.7)
1–6	163 (51.3)	153 (53.1)	316 (52.1)
More than 6	76 (23.9)	61 (21.2)	137 (22.6)
Currently living with parents			
No	84 (26.4)	66 (22.9)	150 (24.8)
Yes	230 (72.3)	221 (76.7)	451 (74.4)
The reason not living with parents			
They are divorced	13 (15.5)	18 (27.3)	31 (20.7)
For my education	30 (35.7)	18 (27.3)	48 (32.0)
My father/mother is not alive	16 (19.0)	9 (13.6)	25 (16.7)
Family economic status			
Good	108 (34.0)	116 (40.3)	224 (37.0)
Fair	123 (38.7)	107 (37.2)	230 (38.0)
Poor	42 (13.2)	27 (9.4)	69 (11.4)

Not all variables add up to 100% because of missing data. ^a^ s.d. = standard deviation

**Table 2:** Health and mental health for participants by sex.

Variables	Total (*n* = 606)	Male (*n* = 318, 52.5%)	Female (*n* = 288, 47.5%)	X^2^	*p*
Self-evaluation of health					
Good	365 (60.2)	204 (64.2)	161 (55.9)	6.984	0.030
Fair	168 (27.7)	74 (23.3)	94 (32.6)		
Poor	6 (1.0)	4 (1.3)	2 (0.7)		
Self-evaluation of academic performance					
Good	373 (61.6)	192 (60.4)	181 (62.8)	1.034	0.596
Fair	188 (31.0)	103 (32.4)	85 (29.5)		
Poor	6 (1.0)	4 (1.3)	2 (0.7)		
Depression (scores)					
None to mild (<10)	311 (51.3)	175 (55.0)	136 (47.2)	3.689	0.055
Moderate to severe (≥10)	295 (48.7)	143 (45.0)	152 (52.8)		
Anxiety (scores)					
None to mild (<10)	483 (79.7)	260 (81.8)	223 (77.4)	1.752	0.186
Moderate to severe (≥10)	123 (20.3)	58 (18.2)	65 (22.6)		
Self-esteem (scores)					
Low (<25)	101 (16.7)	53 (16.7)	48 (16.7)	0.000	1.000
High (≥25)	505 (83.3)	265 (83.3)	240 (83.3)		
Has anybody you know personally done self-harm?					
No	269 (44.4)	132 (41.5)	137 (47.6)	2.561	0.110
Yes	233 (38.5)	131 (41.2)	102 (35.4)		
Has anybody you know personally taken their own life?					
No	492 (81.2)	250 (78.6)	242 (84.0)	7.030	0.008
Yes	48 (8.0)	34 (10.7)	14 (4.8)		
Have you been bullied at school?					
Never	323 (53.3)	161 (50.6)	162 (56.3)	1.067	0.587
Occasionally/sometimes	165 (27.2)	88 (27.7)	77 (26.7)		
Frequently	57 (9.4)	32 (10.1)	25 (8.7)		
Have you bullied others at school?					
Never	385 (63.5)	180 (56.6)	205 (71.2)	11.711	0.003
Occasionally/sometimes	120 (19.8)	77 (24.2)	43 (14.9)		
Frequently	42 (6.9)	24 (7.5)	18 (6.3)		

Not all variables add up to 100% because of missing data.

**Table 3:** Self-harm, suicidal ideation and suicide attempts among participants by sex.

Variables	Total (*n* = 606)	Male (*n* = 318, 52.5 %)	Female (*n* = 288, 47.5%)	X^2^	*p*
Have you ever done self-harm?					
Never	309 (51.0)	163 (51.3)	146 (50.7)	0.268	0.874
Occasionally/Sometimes	204 (33.7)	106 (33.3)	98 (34.0)		
Frequently	65 (10.7)	32 (10.1)	33 (11.5)		
Have you ever thought about taking your own life?					
Never	458 (75.6)	246 (77.4)	212 (73.6)	2.184	0.336
Occasionally/Sometimes	92 (15.2)	42 (13.2)	50 (17.4)		
Frequently	35 (5.8)	17 (5.3)	18 (6.3)		
Have you ever attempted to take your own life?					
Never	528 (87.1)	276 (86.8)	252 (87.5)	0.692	0.708
Occasionally/Sometimes	45 (7.4)	25 (7.9)	20 (6.9)		
Frequently	14 (2.3)	6 (1.9)	8 (2.8)		

**Table 4:** Logistic regression for predictors of self-harm, SI and SA among participants.

Variables	Self-Harm (269, 44.4%)		SI (127, 21.0%) ^a^		SA (59, 9.7%) ^b^	
	COR (95% CI) ^c^	AOR (95% CI) ^c^	COR (95% CI) ^c^	AOR (95% CI) ^c^	COR (95% CI) ^c^	AOR (95% CI) ^c^
Sex						
Male	Ref ^d^	Ref	Ref	Ref	Ref	Ref
Female	1.1 (0.8–1.5)	1.1 (0.7–1.5)	1.3 (0.9–2.0)	1.3 (0.9–2.0)	1.0 (0.6–1.7)	1.0 (0.5–1.7)
Age						
10–14	Ref	Ref	Ref	Ref	Ref	Ref
15–17	3.2 (2.1–5.0) ***	3.6 (2.2–5.9) ***	1.9 (1.2–3.3) *	1.9 (1.1–3.3) *	1.5 (0.7–3.2)	1.9 (0.8–4.6)
18–20	3.8 (2.4–6.2) ***	3.8 (2.3–6.5) ***	1.4 (0.8–2.5)	1.2 (0.7–2.3)	2.4 (1.1–5.1) *	2.6 (1.1–6.5) *
Family economic status						
Good	Ref	Ref	Ref	Ref	Ref	Ref
Fair and poor	1.9 (1.3–2.7) **	1.7 (1.1–2.4) **	1.7 (1.1–2.7) *	1.8 (1.1–2.8) *	1.3 (0.7–2.3)	1.1 (0.6–2.1)
School level						
Middle school student	Ref	Ref	Ref	Ref	Ref	Ref
High school student	3.6 (2.4–5.4) ***	1.9 (0.9–3.7)	2.2 (1.3–3.6) **	1.3 (0.6–2.8)	1.8 (0.9–3.8)	1.5 (0.4–5.0)
Freshman	3.3 (2.0–5.4) ***	1.1 (0.4–2.9)	1.3 (0.7–2.4)	0.7 (0.2–2.0)	2.7 (1.2–6.1) *	1.6 (0.4–7.0)
Religion						
Islam	Ref	Ref	Ref	Ref	Ref	Ref
Christian	1.5 (0.9–2.6)	1.3 (0.7–2.5)	1.2 (0.7–2.3)	1.1 (0.5–2.2)	0.8 (0.3–2.1)	0.5 (0.2–1.8)
Education Level of father						
No education	Ref	Ref	Ref	Ref	Ref	Ref
Madrasah	0.8 (0.3–1.9)	0.7 (0.2–1.8)	0.8 (0.3–2.5)	1.3 (0.4–4.8)	0.6 (0.2–2.2)	1.3 (0.3–5.5)
Primary/middle school	0.8 (0.4–1.9)	0.9 (0.4–2.5)	1.0 (0.4–2.9)	1.7 (0.5–5.6)	0.6 (0.2–1.9)	1.1 (0.3–4.4)
Higher education	0.9 (0.4–2.1)	0.9 (0.4–2.4)	1.2 (0.5–3.0)	1.7 (0.5–5.1)	0.5 (0.2–1.5)	0.9 (0.2–3.2)
Education Level of mother						
No education	Ref	Ref	Ref	Ref	Ref	Ref
Madrasah	0.7 (0.4–1.5)	0.8 (0.4–1.6)	0.4 (0.2–1.1)	0.8 (0.3–2.2)	0.5 (0.2–1.5)	0.9 (0.3–3.5)
Primary/middle school	1.2 (0.7–2.2)	1.4 (0.7–2.7)	0.9 (0.4–1.7)	1.3 (0.6–2.8)	0.7 (0.3–1.6)	1.2 (0.4–3.5)
Higher education	1.0 (0.6–1.8)	1.2 (0.6–2.3)	0.9 (0.5–1.9)	1.5 (0.7–3.2)	0.8 (0.3–1.8)	1.5 (0.5–4.2)
Polygamous father						
No	Ref	Ref	Ref	Ref	Ref	Ref
Yes	0.9 (0.6–1.4)	0.8 (0.5–1.3)	1.1 (0.7–1.8)	1.0 (0.6–1.8)	1.2 (0.6–2.4)	1.0 (0.5–2.1)
Number of father’s children						
1–6	1.0	1.0	1.0	1.0	1.0	1.0
More than 6	0.8 (0.5–1.2)	0.7 (0.4–1.1)	0.7 (0.4–1.2)	0.7 (0.4–1.3)	0.9 (0.4–1.8)	0.9 (0.4–2.0)
Currently living with parents						
No	Ref	Ref	Ref	Ref	Ref	Ref
Yes	0.8 (0.5–1.2)	1.0 (0.6–1.5)	0.7 (0.5–1.2)	0.8 (0.5–1.4)	0.5 (0.3–0.9) *	0.7 (0.4–1.4)
Self-evaluation of health						
Good	Ref	Ref	Ref	Ref	Ref	Ref
Fair and poor	2.0 (1.4–2.9) ***	1.8 (1.2–2.8) **	2.7 (1.8–4.1) ***	2.4 (1.5–3.9) ***	2.1 (1.2–3.7) *	1.8 (0.9–3.5)
Self-evaluation of academic performance						
Good	Ref	Ref	Ref	Ref	Ref	Ref
Fair and poor	1.9 (1.3–2.7) ***	1.5 (1.0–2.3) *	2.0 (1.3–3.0) **	2.0 (1.2–3.1) **	1.8 (1.1–3.2) *	1.8 (0.9–3.4)
Depression (scores)						
None to mild (<10)	Ref	Ref	Ref	Ref	Ref	Ref
Moderate to severe (≥10)	2.4 (1.7–3.4) ***	2.3 (1.6–3.4) ***	2.5 (1.7–3.8) ***	2.3 (1.4–3.5) ***	2.0 (1.1–3.4) *	1.7 (0.9–3.1)
Anxiety (scores)						
None to mild (<10)	Ref	Ref	Ref	Ref	Ref	Ref
Moderate to severe (≥10)	2.4 (1.6–3.7) ***	3.0 (1.8–4.9) ***	2.8 (1.8–4.4) ***	2.8 (1.7–4.5) ***	1.6 (0.9–3.0)	1.5 (0.8–3.0)
Self-esteem (scores)						
Low (<25)	Ref	Ref	Ref	Ref	Ref	Ref
High (≥25)	0.6 (0.4–0.9) *	0.6 (0.4–1.0)	0.4 (0.3–0.7) ***	0.4 (0.3–0.8) **	0.5 (0.3–0.9) *	0.4 (0.2–0.8) *
Has anybody you know personally self-harmed?						
No	Ref	Ref	Ref	Ref	Ref	Ref
Yes	5.9 (4.0–8.7) ***	5.9 (3.8–9.1) ***	2.7 (1.7–4.3) ***	2.6 (1.6–4.2) ***	1.7 (0.9–3.2)	1.6 (0.9–3.1)
Has anybody you know personally taken their own life?						
No	Ref	Ref	Ref	Ref	Ref	Ref
Yes	1.9 (1.0–3.6) *	1.8 (0.9–3.5)	2.4 (1.3–4.5) **	2.6 (1.3–5.3) **	6.2 (3.1–12.4) ***	6.1 (2.8–13.2) ***
Have you been bullied at school?						
Never	Ref	Ref	Ref	Ref	Ref	Ref
Occasionally/sometimes/frequently	2.0 (1.4–2.8) ***	2.0 (1.3–3.0) **	1.8 (1.2–2.6) **	1.7 (1.1–2.7) *	2.1 (1.2–3.7) **	2.2 (1.2–4.0) *
Have you bullied others at school?						
Never	Ref	Ref	Ref	Ref	Ref	Ref
Occasionally/sometimes/frequently	1.4 (0.9–2.0)	1.3 (0.9–2.0)	1.2 (0.8–1.9)	1.4 (0.8–2.2)	1.2 (0.7–2.2)	1.3 (0.7–2.5)

^a^ SI = Suicidal Ideation; ^b^ SA = Suicide Attempts; ^c^ COR: Crude Odds Ratio, AOR: Adjusted Odds Ratio (adjusted for age, sex and family economic status), CI: Confidence Interval; ^d^ Ref means the reference category; *** *p* < 0.001. ** *p* < 0.01. * *p* < 0.05.
